# Implementation of a Quality Improvement and Clinical Decision Support Tool for Cancer Diagnosis in Primary Care: Process Evaluation

**DOI:** 10.2196/65461

**Published:** 2025-06-12

**Authors:** Sophie Chima, Barbara Hunter, Javiera Martinez-Gutierrez, Natalie Lumsden, Craig Nelson, Dougie Boyle, Kaleswari Somasundaram, Jo-Anne Manski-Nankervis, Jon Emery

**Affiliations:** 1 Department of General Practice and Primary Care, Centre for Cancer Research, University of Melbourne, 305 Grattan St, Melbourne, 3010, Australia, 61 410798352; 2 Department of General Practice and Primary Care, University of Melbourne, Melbourne, Australia; 3 Department of Family Medicine, Pontificia Universidad Católica de Chile, Santiago, Chile; 4 Department of Medicine, Western Health, Melbourne, Australia; 5 Primary Care and Family Medicine, LKC Medicine, Nanyang Technological University, Singapore, Singapore

**Keywords:** cancer diagnosis, implementation, clinical decision support tool, diagnosis, primary care, process evaluation, quality improvement, intervention, effectiveness, interviews, surveys, cancer care, general practice

## Abstract

**Background:**

For patients with cancer, the pathway to diagnosis will most often begin in general practice. In the absence of strong diagnostic features or in patients with nonspecific symptoms, delays in diagnosis can occur. Initial presentations and routine blood tests are important in determining whether a patient requires further investigation. Quality improvement interventions, including auditing tools and clinical decision support (CDS), have been developed for use in general practice to support this diagnostic process. We conducted a process evaluation of a pragmatic, cluster-randomized trial that evaluated the effectiveness of a new technology, Future Health Today (FHT), implemented in general practice to assist with the appropriate follow-up of patients at risk of undiagnosed cancer.

**Objectives:**

This study aims to understand implementation gaps, explore differences between the general practices involved, provide context to the trial effectiveness outcomes, and understand the mechanisms behind the intervention successes and failures.

**Methods:**

The trial intervention consisted of the FHT tool (with CDS, audit, recall, and quality improvement components), training and educational sessions, benchmarking reports, and ongoing practice support. The 21 general practices in the intervention arm of the trial were included in the process evaluation. Process data were collected using semistructured interviews, usability and educational session surveys, engagement with intervention components, and technical logs. The Medical Research Council’s Framework for Developing and Evaluating Complex Interventions was used to analyze and interpret the data.

**Results:**

The uptake of the supporting components of the intervention (training and education sessions, benchmarking reports) was low. Most practices only used the CDS component of the tool, facilitated by active delivery, with general practitioners reporting acceptability and ease of use. Complexity, time, and resources were reported as barriers to the use of the auditing tool. Access to a study coordinator and ongoing practice support facilitated the sustained involvement of practices in the trial, while contextual factors, such as the COVID-19 pandemic and staff turnover, impacted their level of participation. The relevance of the intervention varied between practices, with some practices reporting very low numbers of patients who were flagged for further investigation.

**Conclusions:**

While some components of the intervention, such as the CDS tool, were considered to be acceptable and useful, this process evaluation highlighted barriers such as time and resources, practice differences, and considerations around the optimal amount of support needed when delivering the intervention. Addressing these in future studies may optimize the implementation process. Further work is needed to determine if a scaled-back approach, which meets the time and resource availability of a busy general practice, can effectively facilitate the implementation of CDS tools. Given the variation seen between practices, the use of the FHT cancer module may be better targeted to certain practices based on size, location, and patient demographics.

## Introduction

Diagnosing cancer early can improve patient outcomes and quality of life [1,2]. But, in general practice, the timely detection of cancer can be challenging in the absence of strong diagnostic features, often resulting in prolonged diagnostic intervals [3-5]. In patients presenting to general practice with nonspecific symptoms, the use of routine blood tests can guide decision-making [6]. There is strong evidence that supports the diagnostic utility of abnormal blood tests (eg, iron-deficiency anemia and raised platelets) for multiple cancer types [7-9]. However, suboptimal follow-up and management of abnormal test results have been shown to contribute to delays in diagnosis [10].

Inadequate follow-up of abnormal test results may occur in the case of diagnostic errors, but is also influenced by the general practitioners’ (GPs) experience and training; perceptions of cancer care and investigations; patient characteristics; and health system pressures [11,12]. For example, controversy and confusion about prostate-specific antigen (PSA) testing, coupled with changing guidelines and revised thresholds for what is abnormal, contribute to lower follow-up rates in men who have a raised PSA. Surprisingly, there are very few trials that look at modifying the practitioner- and practice-level barriers to following up abnormal results [11].

The general practice electronic medical record (EMR) allows for the integration of novel technologies, where algorithms apply epidemiological data on the underlying risks of undiagnosed cancer based on symptoms and test results to monitor and identify patients who may benefit from further investigation [13]. Clinical decision support (CDS) systems assist in clinical decision-making, where such tools are linked to patient data to produce patient-specific recommendations or prompts for the GP to consider [14,15]. Similarly, auditing tools that use patient information from the EMR enable practice population-level management and review and have the potential to capture patients who are at risk of being lost to follow-up [16,17]. Evidence suggests that tools that highlight patients for review, referral, or further investigation based on evidence-based guidelines can improve patient care, but many of these tools designed to support diagnosis in general practice are met with low uptake and implementation difficulties [18-20].

Complex interventions are used to assess the effectiveness and utility of such tools in general practice. Yet implementing complex interventions can be distinctly difficult, as they involve multiple interrelated components and there are often multiple levels where change is required [21]. Process evaluation can aid in the understanding of the factors that influence how or why a complex intervention succeeds or fails. This study presents the results of a process evaluation of a pragmatic trial, Future Health Today (FHT). This complex intervention consisted of a novel CDS and auditing software, education, quality improvement (QI), and practice support. The pragmatic trial evaluated whether the intervention, which flagged patients with an abnormal blood test that may be indicative of undiagnosed cancer (FHT cancer module), increased the proportion of patients receiving guideline-based care. By gaining process information, we aim to better understand the implementation gaps, explore differences between the general practices involved, understand the interactions between intervention components, and provide context to understand the effectiveness of the intervention.

## Methods

### Intervention Description and Study Population

The FHT study was a pragmatic cluster-randomized controlled trial that evaluated the effectiveness of a QI intervention [22]. Pragmatic trials, by definition, are trials that evaluate an intervention in everyday practice, with the aim of measuring the effectiveness of the intervention in routine clinical practice rather than under ideal conditions [23,24]. The implementation of the FHT software and the trial components (including implementation strategies) were applied and adapted to real-world conditions to understand and evaluate how the tool would be used in routine general practice.

The components of the complex intervention included the FHT software, training and educational sessions, benchmarking reports, and practice support. The trial was conducted between October 2021 and September 2022. Practices were randomly allocated to participate in either the intervention (follow-up of patients with abnormal blood test results associated with the risk of undiagnosed cancer) or the active control (which had access to a different FHT module). As the aim of this process evaluation was to explore the factors critical to the implementation of the cancer module, our study population comprises the 21 intervention arm practices only; results for the active control intervention will be reported separately. The study protocol has been published on the Australia and New Zealand Clinical Trial Registry (ACTRN12620000993998) [25].

**Figure 1.**
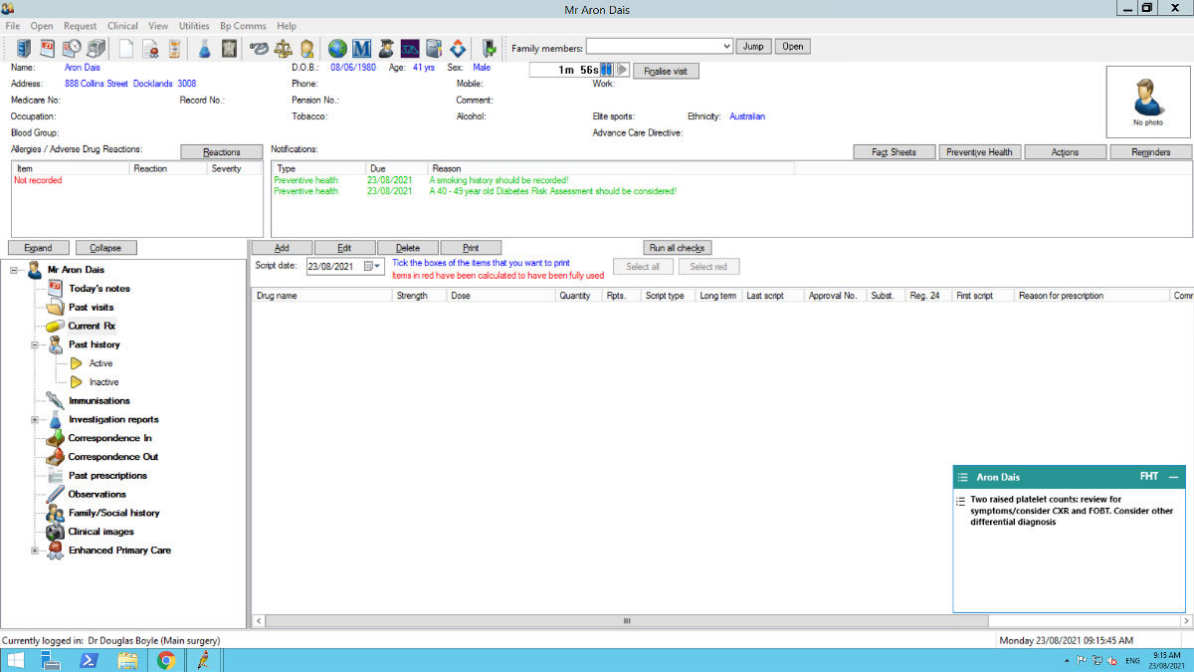
An example of the clinical decision support tool as it appears in the medical record. Simulated patient data are used in this image.

FHT was integrated within the general practice EMR and consisted of a CDS tool, a web-based audit and feedback tool, and the capacity for general practices to monitor their QI activities [26]. Disease-specific modules were developed for use in FHT. The cancer module used patient information in the EMR (age, sex, previous cancer diagnosis) and results of abnormal tests associated with undiagnosed cancers. The FHT cancer module consisted of 3 central algorithms, designed to assist GPs by flagging patients with abnormal blood test results that are associated with an increased risk of undiagnosed cancer: markers of iron deficiency and anemia, raised PSA, and raised platelet count). The CDS component of the tool activates when the GP or general practice nurse (GPN) opens the patients’ medical record, displaying a prompt on screen with guideline-concordant recommendations, such as the review of relevant symptoms or appropriate investigations (Figure 1). There is also a web-based portal, containing an auditing tool; a QI monitoring tool; and access to resources, guidelines, education, and training, which can be accessed on any computer with FHT installed. Algorithms run each night, extracting data from the practice management software database (eg, Best Practice or Medical Director), processing the data locally by applying FHT algorithms (the data does not leave the practice), and categorizing the results. Examples of a CDS prompt and the audit tool are presented in Multimedia Appendix 1. Further details on the development of the tool and the cancer module explored in this study have been described elsewhere [27-29].

In the pragmatic trial, FHT was installed on general practice computers before study initiation. On the first day of the trial, practices were asked to create 3 cohorts of patients using the FHT auditing tool, one for each abnormal blood test (raised PSA, raised platelets, and markers of anemia). The cohorts included all patients identified by the FHT cancer module who had recommendations for guideline-based follow-up (as part of the trial, practices could then review the patient cohorts and determine if further follow-up was necessary). Cohorts were created again at the 6-month mark, using the audit tool, so that benchmarking information could be determined. After generating the cohorts, practices were invited to use FHT as they chose during the trial.

Implementation of the software was supported by a number of additional intervention components. This multifactorial implementation strategy was informed by the Reach, Effectiveness, Adoption, Implementation, and Maintenance framework, with strategies that were relevant and useful to general practice [30]. These components have previously been shown to increase reach; they are low intensity and high impact, with the purpose of limiting implementation workload while promoting continued engagement with the intervention [31,32]. Training on the use of FHT was offered regularly in the lead up to and in the first month of the trial, and then monthly thereafter. Each practice was assigned a study coordinator, who conducted the Zoom-based training sessions on how to use FHT, assisted with any technological queries, and facilitated requests for support throughout the trial. Practices had access to short training videos on YouTube and a range of short- and long-form written training guides. In addition, 6 Project ECHO (Extension for Community Healthcare Outcomes) [33] educational sessions were run on the topics of cancer diagnosis and QI, each consisting of a 10-minute didactic session, a 10-minute case discussion, followed by an open discussion for approximately 20‐30 minutes. The ECHO sessions were delivered via a webinar, and general practice staff were invited to attend. Quarterly benchmarking reports were provided to practices to review their progress in the follow-up of patients who had been flagged by the tool, and to compare their progress to other practices in the trial. All practices were required to nominate a practice champion to lead the implementation of FHT in their practice and to be the primary point of contact with the study coordinator during the trial, managing the installation and technical queries, facilitating ongoing use of the tool, identifying staff for process evaluation interviews and to disseminating trial related information to the practice. The goal of the practice champion in this study was to mirror the pragmatic approach of the intervention (eg, they were asked to filter and disseminate information to the practice using an approach that best reflects their individual practice needs and current processes).

### Ethical Considerations

The study was approved by the Faculty of Medicine, Dentistry and Health Sciences Human Ethics Sub-Committee at the University of Melbourne (ID:2056564). While practices consented on behalf of all practice staff to participate in the wider trial, additional written consent was obtained for all interviews. Interview participants were compensated A $100 (US $64.83) for their time. Practice champions also consented separately and were compensated A $200 (US $129.66) for their role as practice champions. All participant data were deidentified and kept anonymous.

### Data Collection

Data were collected via qualitative interviews, usability surveys, technical queries, engagement logs, and educational session surveys. For the semistructured interviews, all practice champions were contacted via phone and email to participate in an interview in the first and last months of the trial. The practice champion was most commonly a practice manager (PM) or GPN, but GPs occasionally took on this role during the trial (eg, due to staff changes). The semistructured interviews were conducted over the phone. The interviews were conducted by study researchers (SC, NL, and BH; see the following section on researcher characteristics). The duration of the interviews ranged between 15 and 42 minutes. The interview guides were developed using the Clinical Performance Feedback Intervention Theory framework [34] and were pilot-tested during earlier optimization work on the FHT cancer module [27]. The interviews explored installation, intervention delivery, implementation barriers and facilitators, goals, and usability (see Multimedia Appendix 2 for interview schedule). The interviews explored similar themes at each timepoint, although earlier interviews included questions around goals and intention, and the final interviews explored long-term implementation and sustainability. GPs and GPNs were also recruited for interviews in month 6 of the trial. These interviews have been reported separately [35], as the purpose of the clinical interviews was to explore the acceptability of the clinical recommendations and impact on clinical practice, rather than explore the implementation of the wider intervention.

Usability surveys were sent to practice champions in months 1 and 12 of the trial, with the request to distribute them to the rest of the practice. The survey was delivered via web using REDCap (Research Electronic Data Capture; Vanderbilt University) [36] and included 30 questions (multichoice or free text). This survey was anonymous but captured general demographic information about the user and the general practice in which they work. The survey then explored the use and experience with the intervention (eg, length of time using the tool, what components have been used, and feedback and engagement with the intervention components). The survey also included a System Usability Scale (SUS) a 5-point Likert scale that quantifies the perceived usability of FHT [37]. The usability survey was developed by the study implementation team and is available in full in Multimedia Appendix 3).

Postsession ECHO surveys were sent to all ECHO session participants via REDCap after each educational session and collected both demographic information and feedback on the specific learning outcomes of each webinar. The survey consisted of 23 multiple-choice or free-text questions. An example survey from one of the webinars is included in Multimedia Appendix 4.

Information on the number of installations in each practice, the number of individual users, and recommendation queries (submitted through the technology by the practice) was collected using the FHT technology. Technical reports, including any technical queries by the practice throughout the trial, were recorded by the study coordinator. All engagements between the practice and the study team (study coordinator and technical team), were recorded by the study coordinator and categorized by content (eg, technical queries, training, and administrative items). Implementation diaries were kept by study coordinators to record contextual information (eg, changes in COVID-19 pandemic guidelines, immunization rollout, and general practice initiatives) throughout the trial.

### Researcher Characteristics

SC is a PhD candidate at the Department of General Practice and Primary Care, University of Melbourne. BH, a senior qualitative research fellow in the department is the implementation lead for the FHT trial. NL is a postdoctoral research fellow who was the study coordinator for the active control arm of the trial. All are female and experienced in qualitative research and conducting semistructured interviews. Some interview participants were known to the interviewer, given their position in delivering the intervention and supporting the implementation in practices throughout the trial.

### Data Analysis

Recorded interviews were transcribed and imported into NVivo (version 12; Lumivero). Process evaluation data were analyzed independently (SC and BH), prior to trial effectiveness outcomes, so as not to bias the interpretation of the results. Each researcher independently conducted a structured, deductive content analysis of the interview transcripts to extract key themes in the data. The results of the content analysis were collated, and themes were presented to the research team. To promote trustworthiness, analytical codes and emerging concepts and categories were discussed at multiple points in the analysis. Positionality was discussed by the coding team, including how established relationships, biases, and experiences may influence their relationship to the study data, and reflexive notes were kept [38,39]. The interpretation of the key findings and discrepancies in interpretations was discussed with the wider team. The results of the evaluation were then mapped onto the UK Medical Research Council (MRC) framework [40,41].

**Figure 2.**
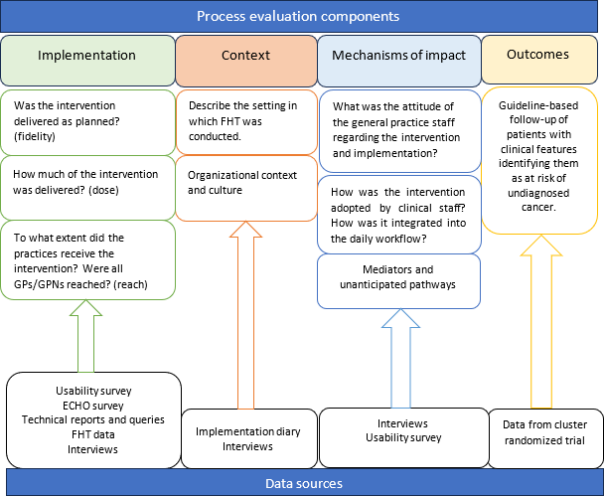
How the process evaluation data are mapped onto the Medical Research Council framework. ECHO: Extension for Community Healthcare Outcomes; FHT: Future Health Today; GP: general practitioner; GPN: general practice nurse.

While several frameworks are available to explore and evaluate the implementation of an intervention, the MRC framework was chosen as it is designed for evaluating complex interventions. It has previously been shown to be useful in evaluating the delivery of new technologies in complex environments and in instances of a multi-faceted implementation approach [42,43]. The framework includes overarching themes of context, implementation, and mechanisms of impact and provides a mechanism for understanding the implementation successes and failures (Figure 2) [40,41]. In the figure, the data sources from the trial are mapped onto the 4 process evaluation components as outlined by the MRC framework (implementation, context, mechanisms of impact, and outcomes). The figure outlines the core components and questions underpinning each theme, and the process data used to answer these questions.

## Results

### Overview

A total of 21 practices participated in the process evaluation. Characteristics of the participating practices are described in Table 1. Characteristics of the interview participants are outlined in Table 2. Participation in other components of the process evaluation (usability survey, ECHO surveys) and additional general practice details are outlined in Multimedia Appendix 5. In summary, 25 interviews were conducted with 19 practice champions in the first and last months of the trial. A total of 12 usability surveys were completed, and 13 post-ECHO session surveys. Usability survey responses included a mix of PMs (n=4), GPNs (n=4), and GPs (n=3), as well as one receptionist (n=1).

**Table 1.** General practice characteristics.

Practice characteristics	Practices (n=21), n (%)
State	
Victoria	20 (95)
Tasmania	1 (5)
Relative Socioeconomic Disadvantage Index (Terciles)	
1 (most disadvantaged)	6 (29)
2	6 (29)
3 (least disadvantaged)	9 (42)
Previously participated in QI^a^ program	9 (43)
Practice size	
4 or fewer FTE^b^ GPs^c^	12 (57)
Greater than 4 FTE GPs	9 (43)
Rurality	
Metro	15 (71)
Rural	6 (29)

a QI: quality improvement.

b FTE: full-time equivalent.

c GP: general practitioner.

**Table 2.** Interview participants by timepoint.

	Month 1	Month 12
Role, n (%)		
GP^a^	1 (7)	1 (9)
GPN^b^	2 (14)	4 (36)
PM^c^	11 (79)	5 (46)
Admin	0 (0)	1 (9)
Gender, n (%)		
Women	13 (93)	11 (100)
Men	1 (7)	0 (0)
Rurality, n (%)		
Metro	11 (79)	5 (45)
Rural	3 (21)	6 (55)
Number of interviewees, n	14	11
Number of practices, n	13	9

a GP: general practitioner.

b GPN: general practice nurse.

c PM: practice manager.

Results have been mapped onto the 3 themes of implementation, context, and mechanisms of impact.

### Trial Results Summary

The results of the cluster randomized controlled trial did not demonstrate a significant improvement in follow-up in the intervention arm [22]. At 12 months, 76.2% (2820/3709) of patients with abnormal test results in the intervention arm had been followed up compared with 70% in the control arm, with an estimated difference of 2.6% (95% CI −2.8% to 7.9%). No significant differences were identified in the secondary analyses or in the time to follow-up of abnormal tests for patients flagged by the tool. The following results of the process evaluation provide some context for the null outcome of the trial and suggest areas for improvement in the development and implementation of CDS and audit software for cancer diagnosis in general practice.

### Implementation

There were 3 core themes on implementation: intervention delivery, installation, and general practice characteristics, each underpinned by different evaluation data sources. Intervention delivery was supported by data from engagement logs and educational session surveys, installation and general practice characteristics were supported by data from technical reports, and all 3 drew from qualitative interview data.

### Intervention Delivery

The intervention consisted of multiple components: the FHT software components (CDS, an auditing tool, and QI monitoring) and the supporting trial components (educational ECHO sessions, zoom-based training sessions, benchmarking reports, and other web-based learning components that practices could opt-in to use). The uptake of the supporting elements of the trial was generally low, except for the initial formal training sessions. GPs, GPNs, and PMs from all intervention practices were invited to the Project ECHO sessions, yet attendance ranged from 2 to 9 people per session, a mix of GPs and GPNs. Three key barriers were assessed as driving the low uptake of these trial components. First, the supporting components of the intervention were promoted via phone calls, newsletters, and regular emails to the practice champion, so it is possible that the knowledge of each session may not have reached the whole practice, dependent on how the practice champion decided to distribute this information to the practice (eg, internal email systems). The second barrier is the time and resource cost associated with each component. For example, attendance at training sessions and ECHO sessions (1 h each), during or after work hours, was not feasible for many clinical staff. The final barrier relates to recognized need and usability, with many practices reporting that they could use the CDS tool and the cancer recommendations adequately, without the need for additional education or training.

*It’s quite straightforward and quite well explained so it didn’t need anything extra particularly*.

[GP, female, month 1]

### Installation

The installation of the software was completed in the month prior to study initiation, with practices having access to a “practice” module on diabetes in the 2 weeks prior to study initiation so any technical issues could be addressed. The installation, which was done remotely and without much interruption to the practice, was reported to be a smooth process for most. For those who required additional assistance, the use of a study coordinator and technical support ensured PMs felt well-supported during this process.

*I think what really has gone well is how it seamlessly was implemented. There was no - there’s no interruption*.

[PM, female, month 1]

Due to the pragmatic approach of the trial, practices determined how many workstations in their practice would have FHT installed at the start of the trial. A total of 14 practices had FHT installed on all clinical computers. Five practices had FHT installed on only one computer at trial initiation, and of these, 4 made the decision to add FHT to additional computers later in the trial. Implementation logs and technical reports indicate that 3 practices were offline for a short period of time (range: 2‐6 wk), although this does not appear to have had a significant impact on the use of the system.

### General Practice Characteristics

There was a large variation in the number of patients identified for follow-up across practices. Three inner-city practices, which had a younger and transient patient population, reported that the cancer module may not be useful in their clinic, given the low number of patients flagged by FHT. For example, in one practice, only 14 patients were flagged for follow-up during the entire 12-month trial period. While these practices acknowledged that the FHT cancer module was less useful for them, it did not deter them from continuing to use the tool after the trial, where they would have access to additional FHT modules (see Software Usability section).

*Actually, it is cancer topic I don’t think that it is very suitable for our clinic because our clinic – the majority of our patients are international students, and they are very young.*

[PM, female, month 12]

### Context

In exploring context, there were 2 prominent themes: the COVID-19 pandemic and staff turnover. Both themes were underpinned by engagement logs, implementation diaries, and qualitative interviews.

### COVID-19

The FHT trial was conducted during the COVID-19 pandemic. In Victoria, restrictions were placed on how and when people could leave their homes, with Melbourne experiencing lockdowns for 262 days during the pandemic. There was a major shift in usual care, and many consultations were conducted via telehealth. During 2020, there was an 8% reduction in cancer-related diagnostic tests nationally, with greater reductions seen in Victoria [44]. The trial continued during a nationwide COVID-19 immunization rollout in primary care, and the burden on general practice was high. There were reports throughout the trial that practices could not devote as much time as they would have liked to FHT or to attend the ECHO sessions due to competing webinars related to COVID-19.

*It’s been a time of change, a lot of updates, a lot of new technology with telehealth. Yeah, there’s been a lot going on because of COVID.*

[PM, male, month 12]

### Staff Turnover

Consequently, staff turnover was a common theme throughout the trial, and the resultant loss of information and increased resource pressure featured heavily in the month 12 interviews. A total of 9 practice champions left their practice during the trial, with 2 practices ending the trial with no replacement. Many interviewees talked about the magnitude of staff turnover during the pandemic and how it was a barrier to use and to keep up momentum in the study.

*We lost two staff, and two doctors at the end of last year. Now we’ve got two doctors that we’re training again. We started off from scratch again.*

[GPN, female, month 12]

### Mechanisms of Impact

We found 4 mechanisms associated with the delivery of the intervention: adoption and integration, training and support, software usability, and clinical recommendations. The sources of data varied within each theme. Technical reports, usability surveys, and interviews supported adoption and integration. Training and support were underpinned by engagement logs, education session surveys, and interviews. Software usability was supported by the usability survey, interviews, and engagement logs. The final theme of clinical recommendations was elucidated from technical reports (in particular, recommendation queries), which were further explored in the educational sessions and qualitative interviews.

### Adoption and Integration

The majority of practices reported that they did not use the QI and audit and recall components of the tool, only the CDS, which was delivered at the point of care. The CDS was considered easy to use and quick to learn and was therefore easily integrated into the clinical workflow by matching the resources available in a busy general practice. However, the audit, recall, and QI components of the tool encountered a number of barriers. First, in comparison to the CDS tool, where recommendations are actively delivered to the GP, the audit and recall tool requires the user to visit a web page and log on to access this part of the tool. Second, there were additional layers of complexity and multiple steps involved in order to identify, review, and recall patients identified in the audit tool.

### Training and Support

The level of engagement between the study coordinator and most participating practices was high, and the support provided by the research and technical team facilitated the continued involvement of practices in the study. No practices in the intervention arm withdrew during the study period.

*The co-operation between the teams and myself was amazing. There were no issues whatsoever and they were always there to help … it was really good.*

[PM, female, month 12]

Practice staff who attended training sessions or used the web-based resources found the training adequate enough to use the tool, and practice champions reported that they would be comfortable training other members of the practice who could not attend. However, in most interviews, especially with the GPs who did not attend the training sessions, it became evident that components of training on how to use FHT did not reach the entire practice. For example, many GPs were unaware of the patient deferral button (which allows GPs to pause recommendations for a patient for a specified period of time) or that there are patient resources available. The post-ECHO session surveys highlighted that the education and case discussion components of the ECHO sessions were useful to GPs and GPNs in managing more complex patient scenarios, but did not influence the way in which the tool was used.

### Software Usability

Of the 12 usability survey responses, 11 (92%) would recommend FHT to others. As part of the usability survey, respondents filled in a SUS [37]. The results of the survey align with the separate qualitative results from the clinical interviews in that FHT is reported to be easy to use, simple, and intuitive [35]. The average SUS score from the respondents was 74 (out of 100), consistent with an above-average score (average score=68; score >70 is considered good).

Acceptance and perceived usefulness of the FHT software were indicated by the number of practices agreeing to continue using the FHT software posttrial. A total of 18 of the 21 practices opted to continue using the software after the trial ended (practices were offered a 3 mo extension), and 17 practices opted to continue using the tool into 2023‐24.

### Clinical Recommendations

The software included a menu option to “report recommendation query” if the GP or GPN thought the recommendation was appearing in error or wanted further information. Five queries about the clinical recommendations in FHT, from 3 practices, were received during the trial. The most frequent recommendation query centered around the clinical recommendations for raised platelets. The risk of undiagnosed cancer increases at a platelet count threshold of 400×10^9^/L, but different laboratories report an upper limit of either 400 or 450 × 10^9^/L; this caused some confusion among GPs if a patient was flagged with a count in the range of 400‐450x 10^9^/L. This issue was addressed in training sessions and regular communications (monthly emails, newsletters), but the perceived error may have impacted some GPs’ willingness to use the tool and their trust in the recommendations. Interestingly, there were no queries about the recommendations for raised PSA (the FHT recommendations were based on current Australian guidelines for PSA follow-up with a lower limit of 3ng/mL in men over 50, which contrasts with some laboratories that report a lower limit of the normal range of 4ng/mL). Established referral pathways and familiarity with the abnormal test as a cancer marker (raised platelet is a relatively new marker of cancer) may have been a contributing factor to this difference in response.

## Discussion

### Overview

In this study, we describe a comprehensive process evaluation exploring the delivery of a complex intervention as part of a pragmatic, randomized trial, where a module to support cancer diagnosis was implemented in general practice. The process evaluation describes implementation gaps and the mechanisms that drive implementation successes and failures in order to provide context to the outcomes from the trial [22].

### Principal Findings

The FHT cancer module intervention did not demonstrate a significant improvement in the follow-up of abnormal test results in the patients flagged by the tool. While we hypothesize that the high-performing practices across both arms may have led to a ceiling effect (ie, there was limited room for improvement given the high rates of follow-up in both arms), an absence of any intervention effect may in part be due to implementation barriers, primarily relating to practice characteristics and contextual factors. There was limited ability for some specific practices to engage with the tool when their patient population was not suited to the FHT module that was implemented. Given this variation in the relevance and usefulness between practices, the use of the FHT cancer module may be better targeted to certain practices based on size, location, and patient demographics.

### Comparison to Prior Work

In comparison to interventions with only one component, complex interventions require more time and resources, and are, unsurprisingly, more difficult to implement [31,45]. We found that the uptake of the supportive components of the intervention was low, aside from some initial training on the software. It was also indicated in the interviews that the supporting components were not considered necessary to use the CDS. While the implementation of new software in general practice requires some training and support, the results of this process evaluation indicate that a scaled-back approach to implementation, one which aligns with the time and resources available to general practice, may have been sufficient for the CDS component of the tool [46]. However, given the null outcomes of the trial, the low uptake of the audit tool, and significant contextual factors (COVID-19 pandemic), more work is needed to determine the usefulness of each component, or combination of components, in supporting this type of change in practice.

Implementing new technologies in general practice is a complex and dynamic process, and despite the potential to improve patient outcomes, many tools have low uptake after implementation [47,48]. The trial consisted of a number of implementation strategies that aimed to optimize the uptake of FHT in routine care, and these methods were applied primarily at the professional level (eg, education or training strategies targeting health care professionals and identification of practice champions) [49]. We found that the use of a practice coordinator facilitated the continued involvement and engagement of practices throughout the trial, similar to previously reported successful implementation strategies used in complex evaluations delivered in general practice. One overview of reviews concluded that practice facilitators, who work with practices in areas such as QI, problem-solving, and education, are almost 3 times as likely to adopt evidence-based guidelines, and practice facilitation improved the adoption of guidelines associated with many chronic diseases [32]. But given the large amount of staff turnover, driven by the COVID-19 pandemic, identifying, maintaining, and replacing practice champions was difficult and resulted in a loss of information and a barrier to engagement for some practices.

### Strengths and Limitations

This process evaluation was extensive, with a multi-modal approach to collecting process data, including interviews, surveys, technical and software data, engagement logs, and implementation diaries. Interviews and usability surveys were carried out at 2 time points during the trial, to address the dynamic nature of implementation barriers and facilitators and how perceptions of the tool can change over time. This substantive evaluation provides context to a complex intervention and the environment in which it was implemented.

There were, however, some limitations. While all practices were invited to take part or contribute to each component of the process evaluation, there were 3 practices who did not participate in an interview at any timepoint or complete any surveys. The opt-in method for the interviews and surveys meant that we may not have sufficiently captured the views of practices who were less engaged with the intervention. These 3 practices did contribute some data to the process evaluation, through software data, technical information, and engagement logs, which were captured from all practices involved in the trial.

The burden of the COVID-19 pandemic in general practice and the resultant impact on staffing was a core theme throughout the process evaluation and provided context when interpreting the trial results. A second limitation was that the pandemic also likely impacted the time, availability, and resources for general practice staff to participate in the interviews and contributed to the low response rate for the usability survey. To mitigate this, we provided numerous opportunities for users to engage in interviews and respond to surveys throughout the trial and promoted such activities through the continued engagement with each practice champion.

Finally, we had originally planned on including some additional software use statistics to complement the qualitative components of the intervention; however, incomplete data prohibited our ability to do so. Software use data would have allowed us to triangulate users’ responses via interviews and surveys with their time using the software, including what parts of the tool they used and when. Future studies would benefit from including software statistics to cross-check the qualitative results.

### Implications and Future Research

There are implications for both research and practice. While the FHT cancer module did not increase the proportion of patients followed up according to guidelines, the process evaluation highlighted factors around usability, which facilitated the adoption and integration of the CDS component of the tool. This, coupled with the acceptability findings from separate clinical interviews [35], and the willingness of the majority of practices to continue using the tool after the trial finished, indicates that different modules developed for use in FHT should be explored, as well as CDS tools for cancer diagnosis more broadly. There are also considerations for designing complex interventions that involve the use of a new technology. Given the low uptake of the supporting components of the tool, but indications of use and acceptability of the CDS component of the software, it is unclear whether a multifaceted implementation strategy is useful when implementing new CDS tools, especially if it has been carefully co-designed to meet the needs of users. Future work should be undertaken to determine if a scaled-back approach, which meets the time and resource availability of general practice, could be as effective in supporting the delivery of novel CDS tools.

### Conclusions

This process evaluation highlights the implementation and process-related gaps that could be addressed in future studies that aim to implement diagnostic support tools for cancer in general practice. While some of the factors were context-specific (eg, driven by the COVID-19 pandemic), barriers such as time, resources, and practice variations, alongside considerations of design elements, could be built upon to optimize future CDS and QI programs.

## Supplementary material

10.2196/65461 Multimedia Appendix 1 Examples of the clinical decision support and audit tool.

10.2196/65461 Multimedia Appendix 2 Example interview guide.

10.2196/65461 Multimedia Appendix 3 Usability survey.

10.2196/65461 Multimedia Appendix 4 Educational session evaluation survey.

10.2196/65461 Multimedia Appendix 5 Characteristics of practices, practice staff, and survey respondents.

## Abbreviations

CDS: clinical decision support
ECHO: Extension for Community Healthcare Outcomes
EMR: electronic medical record
FHT: Future Health Today
GP: general practitioner
GPN: general practice nurse
MRC: Medical Research Council
PM: practice manager
PSA: prostate-specific antigen
QI: quality improvement
REDCap: Research Electronic Data Capture
SUS: System Usability Scale

## References

[1] Pearson C, Fraser J, Peake M (2019). Establishing population-based surveillance of diagnostic timeliness using linked cancer registry and administrative data for patients with colorectal and lung cancer. Cancer Epidemiol.

[2] Swann R, McPhail S, Witt J (2018). Diagnosing cancer in primary care: results from the National Cancer Diagnosis Audit. Br J Gen Pract.

[3] Singh H, Schiff GD, Graber ML, Onakpoya I, Thompson MJ (2017). The global burden of diagnostic errors in primary care. BMJ Qual Saf.

[4] Koo MM, Hamilton W, Walter FM, Rubin GP, Lyratzopoulos G (2018). Symptom signatures and diagnostic timeliness in cancer patients: a review of current evidence. Neoplasia.

[5] Bergin RJ, Emery J, Bollard RC (2018). Rural–urban disparities in time to diagnosis and treatment for colorectal and breast cancer. Cancer Epidemiol Biomarkers Prev.

[6] Pearson C, Poirier V, Fitzgerald K, Rubin G, Hamilton W (2020). Cross-sectional study using primary care and cancer registration data to investigate patients with cancer presenting with non-specific symptoms. BMJ Open.

[7] Bailey SE, Ukoumunne OC, Shephard EA, Hamilton W (2017). Clinical relevance of thrombocytosis in primary care: a prospective cohort study of cancer incidence using english electronic medical records and cancer registry data. Br J Gen Pract.

[8] Mounce LT, Hamilton W, Bailey SE (2020). Cancer incidence following a high-normal platelet count: cohort study using electronic healthcare records from english primary care. Br J Gen Pract.

[9] Yates JM, Logan ECM, Stewart RM (2004). Iron deficiency anaemia in general practice: clinical outcomes over three years and factors influencing diagnostic investigations. Postgrad Med J.

[10] Singh H, Weingart SN (2009). Diagnostic errors in ambulatory care: dimensions and preventive strategies. Adv Health Sci Educ Theory Pract.

[11] Bastani R, Yabroff KR, Myers RE, Glenn B (2004). Interventions to improve follow-up of abnormal findings in cancer screening. Cancer.

[12] Round T, Steed L, Shankleman J, Bourke L, Risi L (2013). Primary care delays in diagnosing cancer: what is causing them and what can we do about them?. J R Soc Med.

[13] Murphy DR, Wu L, Thomas EJ, Forjuoh SN, Meyer AND, Singh H (2015). Electronic trigger-based intervention to reduce delays in diagnostic evaluation for cancer: a cluster randomized controlled trial. J Clin Oncol.

[14] Moja L, Kwag KH, Lytras T (2014). Effectiveness of computerized decision support systems linked to electronic health records: a systematic review and meta-analysis. Am J Public Health.

[15] Dikomitis L, Green T, Macleod U (2015). Embedding electronic decision-support tools for suspected cancer in primary care: a qualitative study of GPs’ experiences. Prim Health Care Res Dev.

[16] Mansell G, Shapley M, Jordan JL, Jordan K (2011). Interventions to reduce primary care delay in cancer referral: a systematic review. Br J Gen Pract.

[17] Nicholson BD, Goyder CR, Bankhead CR (2018). Responsibility for follow-up during the diagnostic process in primary care: a secondary analysis of international cancer benchmarking partnership data. Br J Gen Pract.

[18] Murphy DR, Laxmisan A, Reis BA (2014). Electronic health record-based triggers to detect potential delays in cancer diagnosis. BMJ Qual Saf.

[19] Chima S, Reece JC, Milley K, Milton S, McIntosh JG, Emery JD (2019). Decision support tools to improve cancer diagnostic decision making in primary care: a systematic review. Br J Gen Pract.

[20] Sutton RT, Pincock D, Baumgart DC, Sadowski DC, Fedorak RN, Kroeker KI (2020). An overview of clinical decision support systems: benefits, risks, and strategies for success. NPJ Digit Med.

[21] May C, Finch T, Mair F (2007). Understanding the implementation of complex interventions in health care: the normalization process model. BMC Health Serv Res.

[22] Chima S, Martinez-Gutierrez J, Hunter B (2025). Future Health Today and patients at risk of undiagnosed cancer: a pragmatic cluster randomised trial of quality- improvement activities in general practice. Br J Gen Pract.

[23] Smelt AFH, van der Weele GM, Blom JW, Gussekloo J, Assendelft WJJ (2010). How usual is usual care in pragmatic intervention studies in primary care? an overview of recent trials. Br J Gen Pract.

[24] Roland M, Torgerson DJ (1998). Understanding controlled trials: what are pragmatic trials?. BMJ.

[25] Manski-Nankervis J (2020). Future health today: a cluster randomised controlled trial of quality improvement activities in general practice. Australian New Zealand Clinical Trials Registry.

[26] Hunter B, Biezen R, Alexander K (2020). Future Health Today: codesign of an electronic chronic disease quality improvement tool for use in general practice using a service design approach. BMJ Open.

[27] Chima S, Martinez-Gutierrez J, Hunter B, Manski-Nankervis JA, Emery J (2022). Optimization of a quality improvement tool for cancer diagnosis in primary care: qualitative study. JMIR Form Res.

[28] Hunter B, Davidson S, Lumsden N (2024). Optimising a clinical decision support tool to improve chronic kidney disease management in general practice. BMC Prim Care.

[29] Hunter B, Alexander K, Biezen R (2022). The development of Future Health Today: piloting a new platform for identification and management of chronic disease in general practice. Aust J Prim Health.

[30] Glasgow RE, Harden SM, Gaglio B (2019). RE-AIM planning and evaluation framework: adapting to new science and practice with a 20-year review. Front Public Health.

[31] Lau R, Stevenson F, Ong BN (2015). Achieving change in primary care—causes of the evidence to practice gap: systematic reviews of reviews. Implementation Sci.

[32] Lau R, Stevenson F, Ong BN (2015). Achieving change in primary care--effectiveness of strategies for improving implementation of complex interventions: systematic review of reviews. BMJ Open.

[33] Arora S, Geppert CMA, Kalishman S (2007). Academic health center management of chronic diseases through knowledge networks: project ECHO. Acad Med.

[34] Brown B, Gude WT, Blakeman T (2019). Clinical Performance Feedback Intervention Theory (CP-FIT): a new theory for designing, implementing, and evaluating feedback in health care based on a systematic review and meta-synthesis of qualitative research. Implement Sci.

[35] Chima S, Hunter B, Martinez-Gutierrez J (2024). Adoption, acceptance, and use of a decision support tool to promote timely investigations for cancer in primary care. Fam Pract.

[36] Harris PA, Taylor R, Thielke R, Payne J, Gonzalez N, Conde JG (2009). Research electronic data capture (REDCap)--a metadata-driven methodology and workflow process for providing translational research informatics support. J Biomed Inform.

[37] Lewis JR (2018). The System Usability Scale: past, present, and future. Int J Hum Comput Interact.

[38] Creswell JW, Poth CN (2016). Qualitative Inquiry and Research Design: Choosing among Five Approaches.

[39] Nowell LS, Norris JM, White DE, Moules NJ (2017). Thematic analysis: striving to meet the trustworthiness criteria. Int J Qual Methods.

[40] Moore GF, Audrey S, Barker M (2015). Process evaluation of complex interventions: Medical Research Council guidance. BMJ.

[41] Skivington K, Matthews L, Simpson SA (2021). A new framework for developing and evaluating complex interventions: update of Medical Research Council guidance. BMJ.

[42] Lakshman R, Griffin S, Hardeman W, Schiff A, Kinmonth AL, Ong KK (2014). Using the Medical Research Council framework for the development and evaluation of complex interventions in a theory-based infant feeding intervention to prevent childhood obesity: the baby milk intervention and trial. J Obes.

[43] Bobrow K, Farmer A, Cishe N (2018). Using the Medical Research Council framework for development and evaluation of complex interventions in a low resource setting to develop a theory-based treatment support intervention delivered via SMS text message to improve blood pressure control. BMC Health Serv Res.

[44] The impact of COVID-19 on cancer-related medical services and procedures in australia in 2020. Cancer Australia.

[45] Siebenhofer A, Paulitsch MA, Pregartner G (2018). Cluster-randomized controlled trials evaluating complex interventions in general practices are mostly ineffective: a systematic review. J Clin Epidemiol.

[46] Squires JE, Sullivan K, Eccles MP, Worswick J, Grimshaw JM (2014). Are multifaceted interventions more effective than single-component interventions in changing health-care professionals’ behaviours? An overview of systematic reviews. Implement Sci.

[47] Greenhalgh T, Wherton J, Papoutsi C (2017). Beyond adoption: a new framework for theorizing and evaluating nonadoption, abandonment, and challenges to the scale-up, spread, and sustainability of health and care technologies. J Med Internet Res.

[48] Price S, Spencer A, Medina-Lara A, Hamilton W (2019). Availability and use of cancer decision-support tools: a cross-sectional survey of UK primary care. Br J Gen Pract.

[49] Francke AL, Smit MC, de Veer AJE, Mistiaen P (2008). Factors influencing the implementation of clinical guidelines for health care professionals: a systematic meta-review. BMC Med Inform Decis Mak.

