# Supplementary material for: Implementation of a Quality Improvement and Clinical Decision Support Tool for Cancer Diagnosis in Primary Care: Process Evaluation
Source: JMIR Cancer. 2025 Jun 12;11:e65461. doi: 10.2196/65461 (PMC12178568; doi:10.2196/65461)
Supplement: Multimedia Appendix 1 [file cancer-v11-e65461-s001.pdf]

Multimedia Appendix 1.

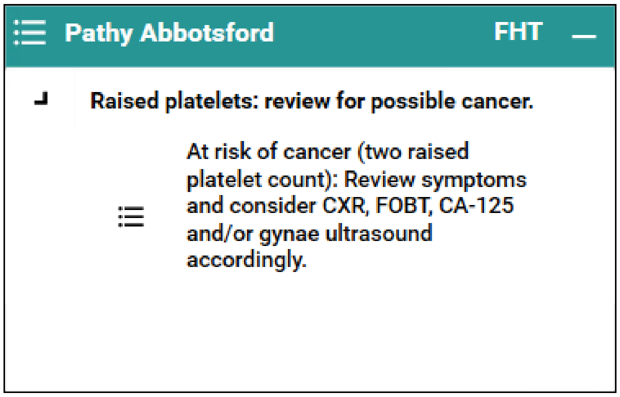

Figure S1. An example of the CDS prompt, with a recommendation for a patient with two consecutive raised platelet counts.

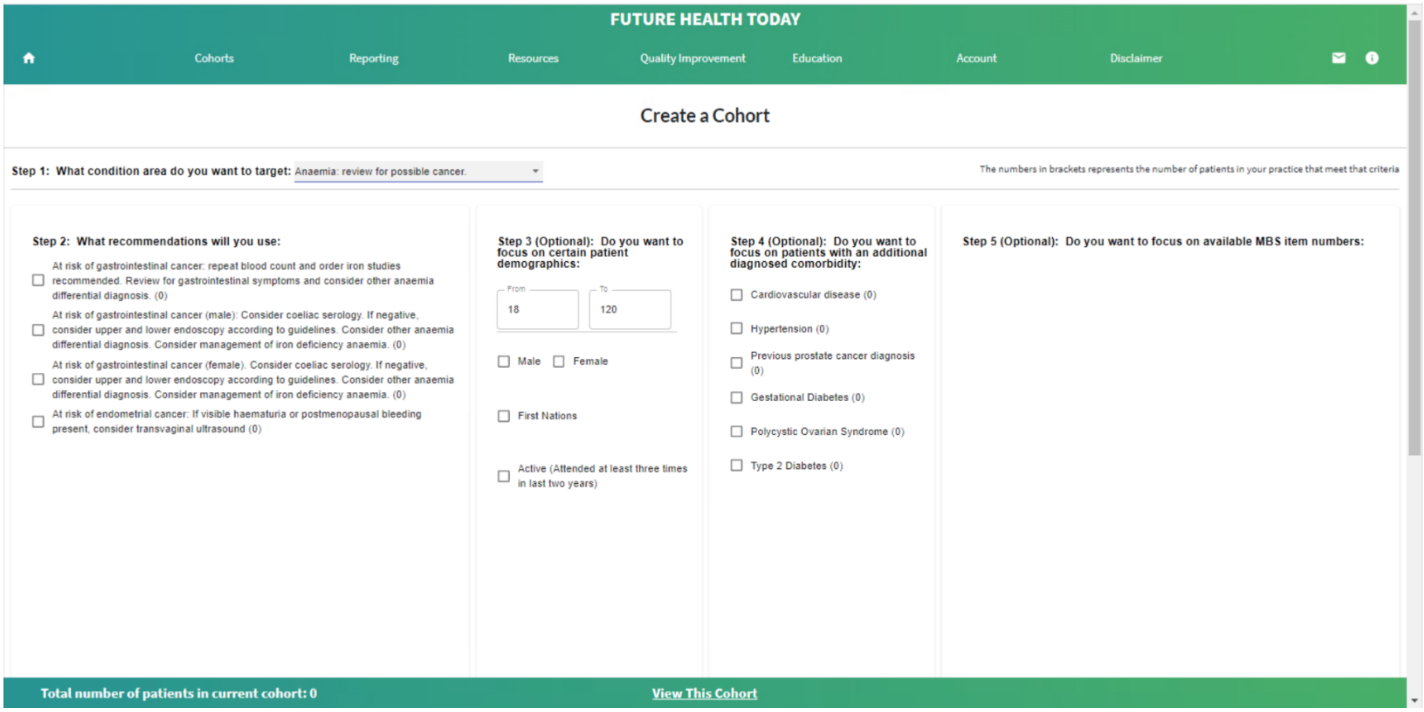

Figure S2. An example of the audit tool, with recommendations for patients with iron-deficiency anaemia
