# Supplementary material for: Implementation of a Quality Improvement and Clinical Decision Support Tool for Cancer Diagnosis in Primary Care: Process Evaluation
Source: JMIR Cancer. 2025 Jun 12;11:e65461. doi: 10.2196/65461 (PMC12178568; doi:10.2196/65461)
Supplement: Multimedia Appendix 2 [file cancer-v11-e65461-s002.pdf]

Multimedia Appendix 2. Example interview guide

Opening question

1. We are now coming to the end of the Future Health Today trial; can you tell me a bit about how things have gone in your practice?
    - *i.e., what has gone well, what hasn't?*
    - *What were the key barriers to use? What were the key facilitators?*
    - *How many staff have been using FHT? Who were the drivers of FHT use in your practice?*

Implementation process

2. What strategies did you use for implementing FHT?
    - *e.g., setting time aside or discussions within your organisation/team?*
    - *What worked well? What didn't?*
3. Did the CPD and QI activities help support implementation and use of FHT?
    - *Were these activities of interest to any/many in the practice? How did you share info about them?*
4. Did the online education sessions (ECHO) help support implementation and use of FHT?
    - *Were staff interested in the sessions?*
    - *How did you share information about the sessions?*
    - *Are there any changes that could be made to make these sessions more appealing to staff, or enable greater participation of staff?*
5. What do you feel you have gained from participating in FHT?
    - *Is being a part of a community of clinical practice and quality improvement important to you and your practice? Do you think FHT facilitated that?*

Point of Care

6. Can you tell me about how you (and/or others) have used FHT at the point-of-care?
    - *How frequently do you use it/do something with it?*
    - *What is your level of confidence using the point-of-care tool?*
    - *Is there anything that helped or hindered your use of the tool (e.g., training)?*
    - *What do you like about the FHT PoC? What do you like the least about the FHT PoC?*

Portal and cohort tool

7. Can you tell me about how you (and/or others) have used the portal and/or cohort tool?
    - *What parts of the portal have you used or accessed/why haven't you accessed the portal?*
    - *Is there anything that helped or hindered this process?*

Training

8. Now you have had FHT in your practice for about 12 months, do you feel confident using it? How long did it take to learn how to use Future Health Today?
    - *Have you needed additional support to use it (from us or within your team)?*

- *What training did you receive/attend and was that level of training sufficient?*
- *Have others in your practice been asking for training or assistance in learning how to use the PoC tool? Or have you had new staff that required additional training? How was this managed?*

Technical issues/technical process

9. Over the past year, can you tell me a bit about your experience with FHT from a technical perspective?
   - *Do you know if you added FHT to any additional computers over the 12 months, or removed FHT from any computers? Have you had any additional installs since the trial started and was it an efficient process?*
   - *Did your practice, you personally or any of your team experience technical difficulties with FHT over the 12 months? What were the nature of these issues, how did they influence your use of FHT, and how did they influence your usual clinical/administrative practice?*
   - *Were these issues resolved and were you satisfied with the resolution?*
   - *Has FHT worked as expected? If not, why?*

Intention/goal/sustainability

10. Now that the trial is finishing, how do you intend to use FHT in your practice?
    - *Has your practice agreed to keep using the tool? Do you want to continue using it/having access to it?*
    - *What were the main drivers/reasons for continuing to use the tool/to stop using the tool?*
    - *Who do you think will lead the use of FHT, now that the trial is finishing?*
