# Supplementary material for: Implementation of a Quality Improvement and Clinical Decision Support Tool for Cancer Diagnosis in Primary Care: Process Evaluation
Source: JMIR Cancer. 2025 Jun 12;11:e65461. doi: 10.2196/65461 (PMC12178568; doi:10.2196/65461)
Supplement: Multimedia Appendix 3 [file cancer-v11-e65461-s003.pdf]

Future Health Today Usability Survey (Month 1)

Thank you for your interest in participating in this research project funded by the Paul Ramsay Foundation, NHMRC and Cancer Research UK. Please take the time to read the plain language statement below before deciding if you would like to participate in this research.

Plain Language Statement What is this research about?
Future Health Today is a technology platform that was designed in consultation with GPs, practice nurses, practice managers, specialist physicians and consumers. The technology aims to assist GPs, practice nurses and practice managers to ensure that people are appropriately screened for chronic diseases, and, if they are found to have a chronic disease, receive the appropriate management.
We want to make sure that the technology we have created is useful and acceptable to health professionals as well as people attending general practice for their medical care. Future Health Today has been implemented in your practice and we would like to know what you think about it.

What will I be asked to do?
We would like you to take 5-10 minutes to complete this online survey so that we can better understand how you use Future Health Today and if you find it accessible and useful. We will also ask you for some demographic and contextual information.

What will happen to information about me?
Your responses to the survey are anonymous and will remain confidential. Your name and the name of your practice will not be recorded. You will not be able to be identified in publications using from this data. Your survey responses will be stored on a secure, password protected server at the University of Melbourne which is only accessible to named researchers and staff. Data collected in this research project may also be used in future projects that are closely related to this project. All data generated during this project will be stored securely for five years from the time of publication arising from use of the data. Data will then be disposed of in keeping with University of Melbourne policy.

Do I have to take part?
No. Participation is completely voluntary however completion of the survey will be considered consent to participate. You are free to terminate your participation at any stage of the project, however any data that has already been collected will not be able to be withdrawn. Your decision to participate will not impact on your relationship with the research group, University of Melbourne or Western Health.

Will I hear about the results of this project?
Results of this study will also be presented at conferences and published on the Data for Decisions website at the University of Melbourne. Your Practice Champion can provide a summary of the results of the research at the conclusion of the study as well as a copy of any publications which result from this research.

Where can I get further information?
If you would like more information about the project, please contact the researchers via e-mail: contact-FHT@unimelb.edu.au or Tel: +61 3 8344 4495

Who can I contact if I have any concerns about the project?
This research project has been approved by the Human Research Ethics Committee of The University of Melbourne. If you have any concerns or complaints about the conduct of this research project, which you do not wish to discuss with the research team, you should contact the Manager, Human Research Ethics, Research Ethics and Integrity, University of Melbourne, VIC 3010. Tel: +61 3 8344 2073 or Email: HumanEthics-complaints@unimelb.edu.au. All complaints will be treated confidentially. In any correspondence please provide the name of the research team (Future Health Today) or the name or ethics ID number of the research project (2056564).

If you agree to participate in this project, please click 'next' to commence the survey.

Please provide name and contact email if you would like to go in the draw for a $100 gift voucher for those that complete the survey ________________________________

1 Gender

◯ Male
◯ Female

2 Age

◯ 21-30
◯ 31-40
◯ 41- 50
◯ 51- 60
◯ 61+

3 Are you a

◯ GP
◯ Practice nurse
◯ Practice manager
◯ Other

Please specify

____

4 How long have you been working in this role?
Please enter number of years.

____

5 Please enter the postcode of your practice

____

6a Please enter approx. number of active patients currently attending your practice

____

6b Please enter approx. number of staff at your practice

____

7 Billing structure

◯ Bulk billing
◯ Mixed billing
◯ Other

Please specify

____

8 EMR used

◯ Medical Director
◯ Best Practice
◯ ZedMed

9 Other than Future Health Today, do you use any systems for quality improvement, audit, or clinical decision support?

☐ PenCAT
☐ POLAR
☐ cdmNET
☐ Canning Tool (Improvement Foundation)
☐ NPS MedicineInsight portal
☐ Doctor's Control Panel
☐ Other

Please specify

____

	Yes (proceed to Q11)	No (proceed to Q12)
10 Have you needed or accessed help or support to use FHT?	◯	◯

11 Who has provided this help or support?

☐ Practice Champion
☐ Colleague
☐ FHT project team
☐ Other

Please specify

____

12 Would you recommend FHT to others?

◯ Yes
◯ No

Please describe why/why not

13 How long have you been using FHT? Please select duration from the dropdown menu

◯ 0-4 weeks
◯ 4-8 weeks
◯ 9-12 weeks
◯ 13-16 weeks
◯ 17-20 weeks
◯ 21-24 weeks
◯ 24-28 weeks
◯ 29-32 weeks
◯ 33-36 weeks
◯ 37-40 weeks
◯ 41-44 weeks
◯ 45-48 weeks
◯ 49- 52 weeks
◯ 52+ weeks

14 Which components have you used?

☐ Point of Care
☐ cohort builder
☐ cohort review
☐ approval
☐ recall
☐ defer
☐ quality improvement
☐ education
☐ reporting
☐ guidelines
☐ training videos
☐ other resources
☐ other components

please specify other resources used

____

please specify other components used

____

15 How many ECHO sessions have you attended?

____

16 Did you find the ECHO sessions helpful?

◯ Yes
◯ No

Please describe why/why not

What did you find helpful?

17 What would you change about the ECHO sessions?

18 Would you recommend the ECHO sessions to others?

◯ Yes
◯ No

19 Please describe why/why not

The System Usability Scale

Please indicate the extent to which you agree or disagree with the following statements (1= strongly disagree, 5 = strongly agree)

	1	2	3	4	5
I think I would like to use this system frequently	○	○	○	○	○
I found the system unnecessarily complex	○	○	○	○	○
I thought the system was easy to use	○	○	○	○	○
I think that I would need the use of a technical person to be able to use the system	○	○	○	○	○
I found the various functions in the system were well integrated	○	○	○	○	○
I thought there was too much inconsistency in this system	○	○	○	○	○
I would imagine that people would learn to use this system very quickly	○	○	○	○	○
I found this system very cumbersome to use	○	○	○	○	○
I felt very confident using the system	○	○	○	○	○
I needed to learn a lot of things before I could get going with this system	○	○	○	○	○

additional comments

	Yes	No
Is there any other comment you would like to make about Future Health Today?	◯	◯

Further comment
