# Supplementary material for: Implementation of a Quality Improvement and Clinical Decision Support Tool for Cancer Diagnosis in Primary Care: Process Evaluation
Source: JMIR Cancer. 2025 Jun 12;11:e65461. doi: 10.2196/65461 (PMC12178568; doi:10.2196/65461)
Supplement: Multimedia Appendix 4 [file cancer-v11-e65461-s004.pdf]

Future Health Today Project ECHO Series: Abnormal test results and cancer risk

Thank you for participating in this ECHO session. Your feedback will be used for evaluation purposes, to optimise future ECHO sessions, and to count towards requirements for continuing professional development points.

Completion of the survey indicates consent for your responses to be used for these purposes

If you would like to claim continuing professional development points, please enter your name	______________________ (Enter Full Name)
and e-mail address	______________________
Please indicate your profession:	◯ GP<br>◯ Practice nurse<br>◯ Other
If other profession please indicate	______________________
If GP, please enter your RACGP or ACRRM number and college	______________________
College	☐ RACGP<br>☐ ACRRM
Practice postcode (please specify)	______________________
Please rate whether learning outcome 1 was met: Describe the risk of undiagnosed cancer in patients with thrombocytosis and the evidence behind it.	◯ Entirely Met<br>◯ Partially Met<br>◯ Not Met
Please rate whether learning outcome 2 was met: Identify some of the pathways to referrals as well as guidelines and resources that can assist you in appropriate follow-up of patients with thrombocytosis at risk of undiagnosed cancer.	◯ Entirely Met<br>◯ Partially Met<br>◯ Not Met
Please rate whether learning outcome 3 was met: Describe adequate follow up and next steps for clinical case considering cancer optimal care pathways when appropriate.	◯ Entirely Met<br>◯ Partially Met<br>◯ Not Met
The didactic topic was relevant to my learning needs	◯ Entirely Relevant<br>◯ Partially Relevant<br>◯ Not Relevant
The content of the didactic presentation was meaningful	◯ Strongly disagree<br>◯ Disagree<br>◯ Neither agree nor disagree<br>◯ Agree<br>◯ Strongly agree

The case discussion was relevant to my learning needs	◯ Entirely Relevant<br>◯ Partially Relevant<br>◯ Not Relevant
There was enough opportunity to contribute to the discussion	◯ Strongly disagree<br>◯ Disagree<br>◯ Neither agree nor disagree<br>◯ Agree<br>◯ Strongly agree
The facilitator managed the discussion appropriately	◯ Strongly disagree<br>◯ Disagree<br>◯ Neither agree nor disagree<br>◯ Agree<br>◯ Strongly agree
The session duration was appropriate for my learning needs	◯ Strongly disagree<br>◯ Disagree<br>◯ Neither agree nor disagree<br>◯ Agree<br>◯ Strongly agree
Overall this activity met my learning needs	◯ Entirely Met<br>◯ Partially Met<br>◯ Not Met
Please rate whether the activity is relevant to general practice	◯ Entirely Relevant<br>◯ Partially Relevant<br>◯ Not Relevant
Overall, I was satisfied with the session	◯ Strongly disagree<br>◯ Disagree<br>◯ Neither agree nor disagree<br>◯ Agree<br>◯ Strongly agree
How might this activity contribute to a systems-based patient safety outcome for your practice? (comment if relevant)	______________________________
Do you have any additional comments?	______________________________
Do you have any suggestions for future ECHO sessions?	______________________________
For the FHT ECHO Series to be successful we require participants to submit cases for discussion. Would you like us to provide you with more information on this to help you consider submitting a case for a future ECHO session?	◯ Yes<br>◯ No

We are currently evaluating the usefulness and accessibility of the ECHO model. If you would like to participate in an interview discussing your thoughts and experience of these ECHO sessions, please provide your name and email address below:

____
