# Supplementary material for: Implementation of a Quality Improvement and Clinical Decision Support Tool for Cancer Diagnosis in Primary Care: Process Evaluation
Source: JMIR Cancer. 2025 Jun 12;11:e65461. doi: 10.2196/65461 (PMC12178568; doi:10.2196/65461)
Supplement: Multimedia Appendix 5 [file cancer-v11-e65461-s005.pdf]

Multimedia Appendix 5.

Table S1. Baseline practice and practice staff characteristics for the intervention arm of the trial

**Practice Characteristics**	N = 21
State	
Victoria	20 (95.2%)
Tasmania	1 (4.8%)
Relative Socio-Economic Disadvantage Index (Terciles)	
1 Most disadvantaged	6 (28.6%)
2	6 (28.6%)
3 Least disadvantaged	9 (42.9%)
Previously participated in QI Program	9 (42.8%)
Practice Size	
4 or fewer FTE GPs	12 (57.1%)
Greater than 4 FTE GPs	9 (42.9%)
Number of FTE GPs[1]	4 (3.5-5.5)
Number of FTE Nurses[1]	2 (1.5-2.5)
Number of FTE Practice Managers/Administrative staff[1]	3.5 (2.5-4.5)
**General practitioners**	145
**Gender**	
Male	85 (58.6%)
Female	60 (41.4%)
**Age**[2]	
< 35 years	21 (15.8%)
35 to 50 years	73 (54.9%)
> 50 years	39 (29.3%)
**Registered nurses**	59
**Gender**	
Male	4 (6.8%)
Female	55 (93.2%)
**Age**[2]	
< 35 years	32 (50%)
35 to 50 years	17 (26.6%)
> 50 years	15 (23.4%)
**Practice managers/ Administrative staff**	127
**Gender**	
Male	13 (10.2%)
Female	114 (89.8%)
**Age**[2]	
< 35 years	55 (43.3%)
35 to 50 years	33 (26.0%)
> 50 years	39 (30.7%)

N – number of practices/practice staff; % - Column percentage; IQR – Interquartile range; FTE – Full-time equivalent; GP - general practitioner.
[1]Median and IQR
[2]Age data not provided for all staff

Table S2. Usability survey demographic details

		n (%)
**Gender**	Female	12 (100%)
	Male	0
**Role**	GP	3 (25%)
	GPN	4 (33%)
	PM	4 (33%)
	Receptionist	1 (8%)
**Rurality**	Metro	8 (67%)
	Rural	4 (33%)
**Age range**	21-30	2 (17%)
	31-40	2 (17%)
	41-50	6 (50%)
	51-60	2 (17%)
**Would you recommend FHT?**	Yes	11 (92%)
	No	1 (8%)

Table S3. ECHO survey respondent demographic details

		n (%)
**Gender**	Female	6 (75%)
	Male	2 (25%)
**Role**	GP	5 (63%)
	GPN	2 (25%)
	PM	1 (12%)
**Rurality**	Metro	2 (25%)
	Rural	6 (75%)
